# Caesarean section and its relationship to offspring general cognitive ability: a registry-based cohort study of half a million young male adults

**DOI:** 10.1136/ebmental-2021-300307

**Published:** 2021-09-12

**Authors:** Viktor H Ahlqvist, Lucas D Ekström, Egill Jónsson-Bachmann, Per Tynelius, Paul Madley-Dowd, Martin Neovius, Cecilia Magnusson, Daniel Berglind

**Affiliations:** 1 Department of Global Public Health, Karolinska Institutet, Stockholm, Sweden; 2 Clinical Epidemiology Division, Department of Medicine (Solna), Karolinska Institutet, Stockholm, Sweden; 3 Department of Public Health and Caring Sciences, Uppsala Universitet, Uppsala, Sweden; 4 Centre for Epidemiology and Community Medicine, Region Stockholm, Stockholm, Sweden; 5 Centre for Academic Mental Health, Population Health Sciences, Bristol Medical School, University of Bristol, Bristol, UK

## Abstract

**Background:**

A relationship between caesarean section and offspring cognitive ability has been described, but data are limited, and a large-scale study is needed.

**Objective:**

To determine the relationship between mode of delivery and general cognitive ability.

**Methods:**

A cohort of 579 244 singleton males, born between 1973 and 1987 who conscripted before 2006, were identified using the Swedish population-based registries. Their mode of delivery was obtained from the Swedish Medical Birth registry. The outcome measure was a normalised general cognitive test battery (mean 100, SD 15) performed at military conscription at around age 18.

**Findings:**

Males born by caesarean section performed poorer compared with those born vaginally (mean score 99.3 vs 100.1; adjusted mean difference −0.84; 95% CI −0.97 to −0.72; p<0.001). Both those born by elective (99.3 vs 100.2; −0.92; 95% CI −1.24 to −0.60; p<0.001) and non-elective caesarean section (99.2 vs 100.2; −1.03; 95% CI −1.34 to −0.72; p=0.001), performed poorer than those born vaginally. In sibling analyses, the association was attenuated to the null (100.9 vs 100.8; 0.07; 95% CI −0.31 to 0.45; p=0.712). Similarly, neither elective nor non-elective caesarean section were associated with general cognitive ability in sibling analyses.

**Conclusion:**

Birth by caesarean section is weakly associated with a lower general cognitive ability in young adult males. However, the magnitude of this association is not clinically relevant and seems to be largely explained by familial factors shared between siblings.

**Clinical implication:**

Clinicians and gravidas ought not to be concerned that the choice of mode of delivery will impact offspring cognitive ability.

## Background

Delivery by caesarean section has surged globally, with an increase from 7% to 19% of all births between 1990 and 2014.^1 2^ Although caesarean section is a vital intervention under specific obstetric circumstances, its use now well exceeds the recommended population level of 15%.^3^ Alarmingly, in some countries (eg, Brazil and Egypt), more children are now born by caesarean section than vaginally.^2^ This is particularly notable since access to this delivery method varies between countries and is unequally distributed according to socioeconomic factors within countries.^1^

The increase in caesarean sections has sparked concerns and interest in whether the intervention may carry long-term health risks for children.^4–7^ Caesarean section has repeatedly been implicated in offspring atopic,^6 7^ autoimmune,^8^ metabolic,^5^ neurological^4^ and psychiatric conditions.^4^ However, as we have previously shown for offspring obesity^9^ and offspring cardiorespiratory fitness^10^ and others for neuropsychiatric conditions,^4^ these relationships may be driven by unobserved confounding factors, such as those shared between siblings.^4^

Nevertheless, few studies have examined the relationship between caesarean section and offspring cognitive ability,^11 12^ a factor with substantial influence on offspring long-term health.^13 14^ The few available studies of caesarean section and offspring cognitive ability have focused on children and have been inconsistent due to widespread outcome measure heterogeneity.^12 15 16^ Specifically, previous studies of cognitive outcomes have often relied on proxy measures of cognitive ability (eg, school performance or parent-reported ability), warranting further investigation. Furthermore, no previous study of measured cognitive ability has used family-based methods to account for unobserved confounders shared within families (genetic and environmental),^12^ although a recent paper employed sibling analysis in the study of educational performance.^17^

Despite the aforementioned challenges, a series of possible mechanisms for a relationship between birth by caesarean section and offspring morbidity later in life have been suggested, for example, microflora colonisation,^18^ stress mechanisms,^19^ DNA methylation^20^ and hormonal effects.^19^ The relevance of these mechanisms/exposures may, however, vary with the type of caesarean section. That is, exposure to the assumed beneficial stress of labour would differ according to whether a caesarean section is performed prelabour or postlabour onset. Yet, few studies have relied on structured reporting of the type of caesarean section,^12^ impacting their ability to reliably discriminated between elective and non-elective caesarean section and limiting the appreciation of possible causal effects.

## Objective

Here, we aim to fill these knowledge gaps and determine the relationship between caesarean section and offspring general cognitive ability. Taking advantage of a large, population-based sample with professional assessments of cognitive ability and novel family-based methods, we investigate whether elective or non-elective caesarean sections are differentially associated with general cognitive ability, and to what extent these associations can be explained by unobserved confounding shared within families.

## Methods

### Patient and public involvement statement

All analyses used pre-existing data from health registries and, as such, neither patients nor the public was involved in the study design, data collection and analysis, interpretation of findings, decision to publish or preparation of the manuscript.

### Study design: data sources

We identified all singleton males born in Sweden between 1973 and 1987. Using the unique Swedish personal identity number for linkage of population-based registers, we collected birth and pregnancy data from the Swedish Medical Birth Registry,^21^ parental sociodemographic data from the population and housing censuses,^22^ familial-linkage information from the multigenerational registry^22^ and conscription information from the Swedish conscription registry.^23^

### Derivation of the study population

**Figure 1:**
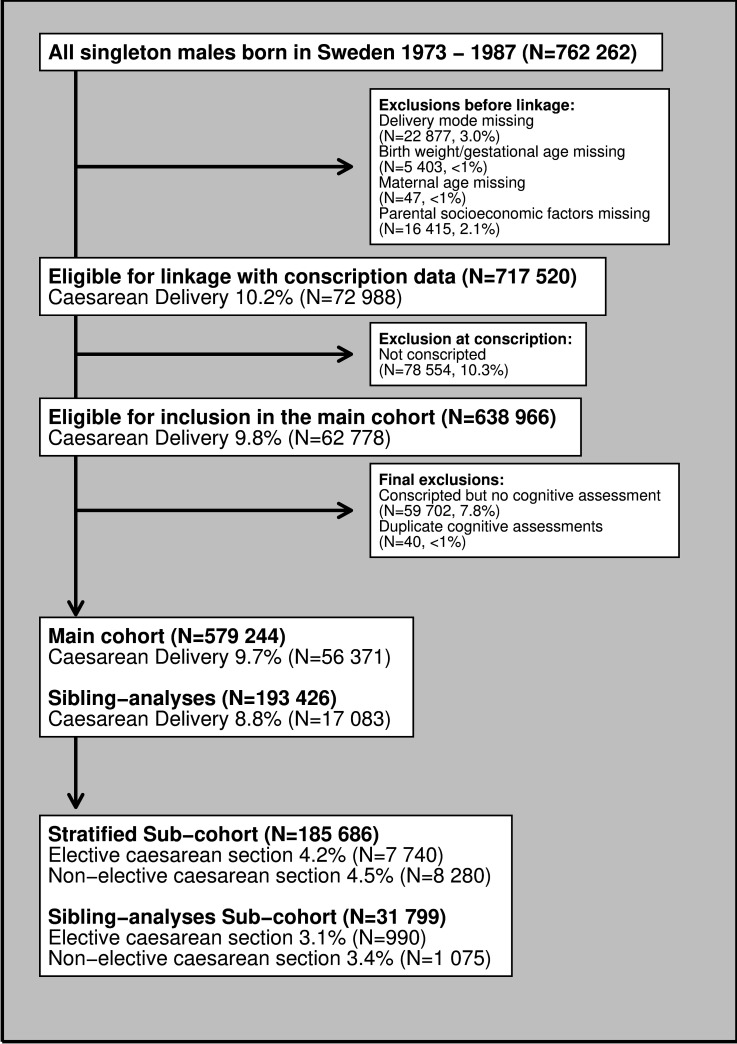
Flow chart of the derivation of the main and subcohort.

Among all singleton males identified in the Medical Birth Registry (N=762 262), covering 99% of the Swedish born population (figure 1),^21^ we excluded those with missing information on the mode of delivery (N=22 877, 3.0%), birthweight and gestational age (N=5 403,<1%), maternal age (N=40,<1%), or parental socioeconomic factors (N=16 415, 2.1%). We then excluded those not conscripted before 2006 (N=78 554, 10.3%) (including, as constituted by law, individuals with severe medical conditions) and those not participating in the cognitive assessments at conscription (N=59 702, 7.8%) or having duplicate recorded scores (N=40, <1%) (online supplemental table 1).

10.1136/ebmental-2021-300307.supp1 Supplementary data

This resulted in a main cohort of 579 224 conscripts (76% retained). A subset of these, N=193 426 were full brothers that formed the study population used for our sibling analyses (online supplemental table 2).^24 25^ Further, we defined yet another subset of the main cohort including conscripts born between 1982 and 1987, N=185 686, in whom detailed delivery data including type of caesarean section was available.

### Exposure: mode of delivery

Mode of delivery was obtained from the Medical Birth Registry, which was routinely collected throughout the study period with good quality.^26^ The primary exposure was dichotomised as either (1) vaginal delivery (with or without instrumental support) or (2) caesarean delivery.

In a subset of the main cohort (figure 1), born between 1982 and 1987, data availability allowed us further to trichotomise mode of delivery into a secondary exposure: (1) vaginal delivery (with or without instrumental support), (2) elective caesarean section (defined as a prelabour caesarean section) or (3) non-elective caesarean section (defined as postonset of labour caesarean section).

### Outcome: general cognitive ability

The Swedish Conscription Agency has employed cognitive assessments since 1944,^23^ intending to determine conscript cognitive capacity to suitably match military positions. For the time period when the individuals in the cohort were conscripted, two different test batteries were used to estimate the general intelligence factor (G-factor): Between 1991 and 1996 the Swedish Enlistment Battery 80 (SEB80) was used, followed by the Computer-Aided Testing Swedish Enlistment Battery (CAT-SEB) in 1997 through 2005. The SEB80 consisted of four paper-and-pencil subtests assessing logical, technical, verbal and visuospatial abilities.^27^ The similar, but computerised, CAT-SEB consisted of 12 tests, 10 of which were used to construct a general cognitive ability factor.^28^ The reliability and construct validity of both the SEB80 and the CAT-SEB are high.^27 28^ Specifically, both the SEB80 and CAT-SEB have been thoroughly evaluated for their ability to identify latent cognitive factors, with prior studies demonstrating that the tests have robust construct validity for a general intelligence factor while decomposed factors are less robust (eg, verbal ability and spatial ability).^27 28^ Furthermore, CAT-SEB correlates well with the Swedish Scholastic Aptitude Test, with the crystallised intelligence factor of the CAT-SEB being especially dominant in the scholastic test.^29^

The scores from both the SEB80 and CAT-SEB were standardised for each conscription year by the Swedish Conscription Agency to stanine scores, that is to follow a normal distribution with nine levels (mean=5 and SD=2). To enhance comparability with other studies using an IQ test to estimate cognitive ability, we transformed the stanine scores to a mean of 100 with an SD of 15. Except for in sensitivity analyses, we treated the cognitive assessments as synonymous, that is, we obtained whichever test the conscript performed, hereafter referred to as the general cognitive ability assessment.

### Covariates

We considered a series of putative confounders, all of which have been implicated as determinants for mode of delivery and offspring health. Specifically, we controlled for the following maternal factors: maternal age at delivery (continuous),^30^ ^s31^ pre-eclampsia(yes/no) (ICD-8: 637,03–637,10 and ICD-9 642E-642G),^s32 s33^ pregestational hypertension (yes/no)^s32 s33^ and pre-existing or early-pregnancy diabetes mellitus (yes/no).^s34 s35^ We additionally controlled for the following sociodemographic factors^1^ ^s36^: family disposable income (quintiles), parental country of birth (both foreign born, one parent born in Sweden or both born in Sweden), highest parental education (primary, secondary or university level), and parental labour market position (non-manual high, non-manual intermediate, non-manual low, farmers, skilled workers, unskilled workers and other). Disposable income and labour market position was obtained for every fifth year between 1970 and 1990, and retained from the census preceding the birth of the child to reduce possible collider stratification. Parental education was recorded in 1970 and 1990, and we retained the highest ever recorded level of education to capture both socioeconomic position and parental scholastic aptitude. Further, we adjusted all analyses for offspring year of birth (continuous, treated as categorical) and maternal parity (continuous, treated as categorical).^s37 s38^ Finally, except for in a certain sensitivity analysis, we controlled for the following fetal characteristics: birth weight z-score (continuous)^s39 s40^ internally standardised by gestational week, and gestational age (continuous).^s30 s41^

### Statistical analyses

We present descriptive characteristics of the main and sub-cohort, stratified by the primary exposure, using measures of central tendency and dispersion. We employed linear regression to assess the relationship between mode of delivery (for both primary and secondary exposure) and general cognitive ability. For sibling analyses, we employed linear fixed-effects regressions, which holds all family-specific factors (shared factors) constant using the demeaning approach.^s42^ As such, the fixed-effects regression yields an estimate of the within-effect, accounting for all factors shared between brothers (genetic and environmental).

In explorative efforts to identify pregnancies where a caesarean section has a differential impact, we repeated our main adjusted model stratifying by gestational periods (extremely preterm, very preterm, moderate to late preterm, early term, full term, late term, post-term),^s43^ birth year (grouped by every 2 years), parity (1, 2, 3, 4+), maternal age (15-, 20-, 25-, 30-, 35-, 40-), highest parental education, household disposable income quintile and a modified Robson classification (1 & 2, 3 & 4, 5, 6 & 7, 9 & 10) previously used in the Nordic countries^s44^—excluding multifetal gravidas (class 8) (online supplemental table 3). For each stratified analysis, we excluded adjustment for the strata specific confounder.

For all analyses, we provide unadjusted and confounder adjusted results. In sibling analyses, we control for all shared factors (observed and unobserved) and further control for observed non-shared factors (ie, the putative confounders, excluding parental sociodemographic factors which did not vary). To account for the correlation between brothers we used robust (sandwich) SE estimation for all analyses. All analyses were conducted using Stata V.15.1.

### Sensitivity analyses

We performed a series of sensitivity analyses to scrutinise our main analysis. First, we repeated our main analysis stratified by the type of battery that the conscripts performed. Further, we decomposed the SEB80 into technical, logical, verbal and visuospatial comprehension, and the CAT-SEB into verbal ability (crystallised intelligence) and spatial ability (general visualisation).

Second, as those with severe disabilities are ineligible for conscription, it is plausible that such exclusion results in a healthy participant selection (bias towards the null). Therefore, we analyse the relationship between caesarean section and prematurity (<37 completed weeks of gestation), which we believe to be causal under iatrogenic early delivery.^s45^ Beyond the assumption that the aforementioned relationships are causal, we assume that the pattern of selection bias is consistent between the outcomes cognitive ability and prematurity (both to null by healthy selection).^s46^ Under these assumptions, this can be considered a positive control outcome scenario—where a null association between caesarean section and prematurity suggests a persistent bias towards the null in all analyses of the conscription cohort.

Third, to relax homogeneity assumptions of vaginal deliveries, we separated the vaginal deliveries into instrumental and non-instrumental. Forth, in efforts to control for previous caesarean section in the mother, we repeated our main analysis while controlling for any maternal history of caesarean section, using available information from in the Medical Birth Registry. We further repeated our main analyses, adjusting for maternal smoking (non-smoker, 1–9 cigarettes/day,≥10 cigarettes/day),^s47 s48^ maternal early pregnancy body mass index (BMI) (continuous, with a cubic term),^s49 s50^ and gestational weight gain^s51^ standardised to the week of gestation and early pregnancy BMI using Swedish reference values.^s52^

Fifth, in the main analysis we controlled for gestational age and birth weight z-score, which under certain causal pathways may act as a collider (online supplemental figure 1). Therefore, we repeat our main analysis excluding adjustment for gestational age and birth weight z-score. Finally, as previous validity reports have noted that there may be some misclassification of caesarean section type in those born preterm,^21^ we repeated our main and sub-analysis restricted to term births.

## Findings

### Descriptive characteristics

Among the included male conscripts, 9.7% (56 371/579 244) were born by caesarean section (table 1). Those born vaginally had a higher mean birth weight (3 601 grams) as compared with those born by any type of caesarean section (3 372 grams), while males delivered by caesarean section were more likely to be born prematurely (1.4% vs 0.2%) and small for gestational age (14.2% vs 9.2%). Further, maternal pre-eclampsia (1.9% vs 0.5%), gestational hypertension (0.3% vs 0.1%), diabetes mellitus (1.8% vs 0.3%) and a higher mean maternal age (29.0 vs 27.5 years), was more common in caesarean than vaginal births. Lastly, parents of those born by caesarean section had a higher education, family disposable income and labour market position.

**Table 1:** Descriptive characteristics of the main cohort and the subcohort with detailed delivery data, stratified by mode of delivery

	Main cohort		Subcohort with detailed delivery data		
	Vaginal delivery	Any caesarean section	Vaginal delivery	Elective caesarean section	Non-elective caesarean section
Total no of observations, (%)	522 853 (90.3)	56 371 (9.7)	169 666 (91.4)	7 740 (4.2)	8 280 (4.5)
Age at conscription (years), median (IQR)	18.3 (18.1–18.5)	18.3 (18.1–18.5)	18.3 (18.1–18.5)	18.3 (18.1–18.5)	18.3 (18.1–18.5)
Birth weight (g), mean (SD)	3 600.8 (516.0)	3 371.9 (677.1)	3 617.3 (512.8)	3 410.3 (575.0)	3 309.1 (813.7)
Fetal growth, no (%)					
Small for gestational age (<10th %ile)	48 141 (9.2)	7 981 (14.2)	12 569 (7.4)	725 (9.4)	1 271 (15.4)
Appropriate for gestational age	421 966 (80.7)	42 263 (75.0)	138 217 (81.5)	5 997 (77.5)	6 061 (73.2)
Large for gestational age (>90th %ile)	52 746 (10.1)	6 127 (10.9)	18 880 (11.1)	1 018 (13.2)	948 (11.4)
Gestational age (weeks), mean (SD)	39.7 (1.7)	38.7 (2.3)	39.5 (1.6)	38.1 (1.5)	38.5 (3.0)
Gestational periods, no (%)					
Extremely preterm (<28 wGA)	170 (<1)	54 (0.1)	51 (<1)	<10	29 (0.4)
Very preterm (28–31 wGA)	1 027 (0.2)	809 (1.4)	248 (0.1)	42 (0.5)	277 (3.3)
Moderate to late preterm (32–36 wGA)	18 953 (3.6)	6 066 (10.8)	6 594 (3.9)	506 (6.5)	1 424 (17.2)
Early term (37–38 wGA)	77 017 (14.7)	16 996 (30.2)	28 678 (16.9)	4 723 (61.0)	1 589 (19.2)
Full term (39–40 wGA)	263 153 (50.3)	21 725 (38.5)	89 969 (53.0)	2 116 (27.3)	2 672 (32.3)
Late term (41 wGA)	104 998 (20.1)	5 798 (10.3)	31 511 (18.6)	209 (2.7)	1 337 (16.1)
Post-term (≥42 wGA)	57 535 (11.0)	4 923 (8.7)	12 615 (7.4)	143 (1.8)	952 (11.5)
Modified Robson classification, no (%)					
1+2	203 564 (38.9)	22 112 (39.2)	64 605 (38.1)	1 755 (22.7)	3 912 (47.2)
3+4	287 984 (55.1)	16 654 (29.5)	94 088 (55.5)	2 620 (33.9)	1 791 (21.6)
5	4 324 (0.8)	5 979 (10.6)	2 718 (1.6)	2 068 (26.7)	440 (5.3)
6+7	5 803 (1.1)	5 366 (9.5)	985 (0.6)	781 (10.1)	601 (7.3)
9+10	21 178 (4.1)	6 260 (11.1)	7 270 (4.3)	516 (6.7)	1 536 (18.6)
Parity, median (IQR)	2.0 (1.0–2.0)	2.0 (1.0–2.0)	2.0 (1.0–2.0)	2.0 (1.0–3.0)	1.0 (1.0–2.0)
Maternal age at birth (years), mean (SD)	27.5 (4.9)	29.0 (5.6)	28.3 (5.0)	30.5 (5.4)	28.7 (5.4)
Categories of maternal age, no (%)					
<20 years	24 507 (4.7)	1 960 (3.5)	5024 (3.0)	98 (1.3)	235 (2.8)
20–24 years	147 504 (28.2)	12 554 (22.3)	41 037 (24.2)	1 133 (14.6)	1 972 (23.8)
25–29 years	198 443 (38.0)	19 087 (33.9)	63 567 (37.5)	2 538 (32.8)	2 977 (36.0)
30–34 years	113 144 (21.6)	13 936 (24.7)	42 281 (24.9)	2 260 (29.2)	1 968 (23.8)
≥35 years	39 255 (7.5)	8 834 (15.7)	17 757 (10.5)	1 711 (22.1)	1 128 (13.6)
Maternal diabetes mellitus, no (%)	1 578 (0.3)	987 (1.8)	871 (0.5)	173 (2.2)	95 (1.1)
Maternal hypertension, no (%)	449 (0.1)	165 (0.3)	330 (0.2)	44 (0.6)	48 (0.6)
Pre-eclampsia, no (%)	2 437 (0.5)	1 046 (1.9)	2 046 (1.2)	212 (2.7)	505 (6.1)
Highest parental education, no (%)					
Primary education	67 918 (13.0)	7 415 (13.2)	17 184 (10.1)	810 (10.5)	890 (10.7)
Secondary education	262 833 (50.3)	27 155 (48.2)	86 717 (51.1)	3 721 (48.1)	4 257 (51.4)
University education	192 102 (36.7)	21 801 (38.7)	65 765 (38.8)	3 209 (41.5)	3 133 (37.8)
Familial disposable income, no (%)					
Quintile 1	60 174 (11.5)	6 963 (12.4)	35 421 (20.9)	1 445 (18.7)	1 829 (22.1)
Quintile 2	104 135 (19.9)	10 709 (19.0)	42 007 (24.8)	1 709 (22.1)	2 056 (24.8)
Quintile 3	124 926 (23.9)	12 251 (21.7)	36 535 (21.5)	1 647 (21.3)	1 814 (21.9)
Quintile 4	120 651 (23.1)	12 696 (22.5)	30 646 (18.1)	1 481 (19.1)	1 394 (16.8)
Quintile 5	112 967 (21.6)	13 752 (24.4)	25 057 (14.8)	1 458 (18.8)	1 187 (14.3)
Parent(s) born in Sweden, no (%)					
Both parents	452 640 (86.6)	48 063 (85.3)	145 352 (85.7)	6 571 (84.9)	6 983 (84.3)
One parent	49 343 (9.4)	5 854 (10.4)	16 503 (9.7)	837 (10.8)	854 (10.3)
Neither parent	20 870 (4.0)	2 454 (4.4)	7 811 (4.6)	332 (4.3)	443 (5.4)
Parental labour market position, no (%)					
Others	19 521 (3.7)	2 283 (4.0)	7 417 (4.4)	348 (4.5)	368 (4.4)
Unskilled workers	93 311 (17.8)	9 386 (16.7)	26 995 (15.9)	1 164 (15.0)	1 374 (16.6)
Skilled workers	101 566 (19.4)	10 170 (18.0)	32 423 (19.1)	1 328 (17.2)	1 576 (19.0)
Self-employed and farmers	32 069 (6.1)	3 586 (6.4)	10 090 (5.9)	440 (5.7)	484 (5.8)
Non-manual workers at lower level	78 417 (15.0)	8 481 (15.0)	24 577 (14.5)	1 141 (14.7)	1 256 (15.2)
Non-manual workers at intermediate level	126 455 (24.2)	13 620 (24.2)	40 956 (24.1)	1 872 (24.2)	1 932 (23.3)
Non-manual workers at higher level	71 514 (13.7)	8 845 (15.7)	27 208 (16.0)	1 447 (18.7)	1 290 (15.6)

%ile, percentile; IQR, interquartile range; No., number of observations; SD, standard deviation; wGA, weeks of gestational age.

In the subcohort where we stratified by type of caesarean section, we observed similar patterns of characteristics as those in the main cohort; gravidas receiving either type of caesarean section were more likely to suffer from morbidity as compared with their vaginally delivering peers. Further, as expected, those born by non-elective caesarean section had the highest occurrence of pre-eclampsia (6.1%) while those born by elective caesarean section had the highest rates of maternal diabetes mellitus (2.2%). Notably, those born by elective caesarean section had the highest familial socioeconomic position of all groups including the highest parental education, family disposable income, and parental labour market position. Finally, mothers of those born by elective caesarean section were older (mean 30.5 years at birth), while those of those born by non-elective caesarean section were comparable to those born vaginally (mean 28.3 and 28.7 years, respectively).

### Main analyses

**Figure 2:**
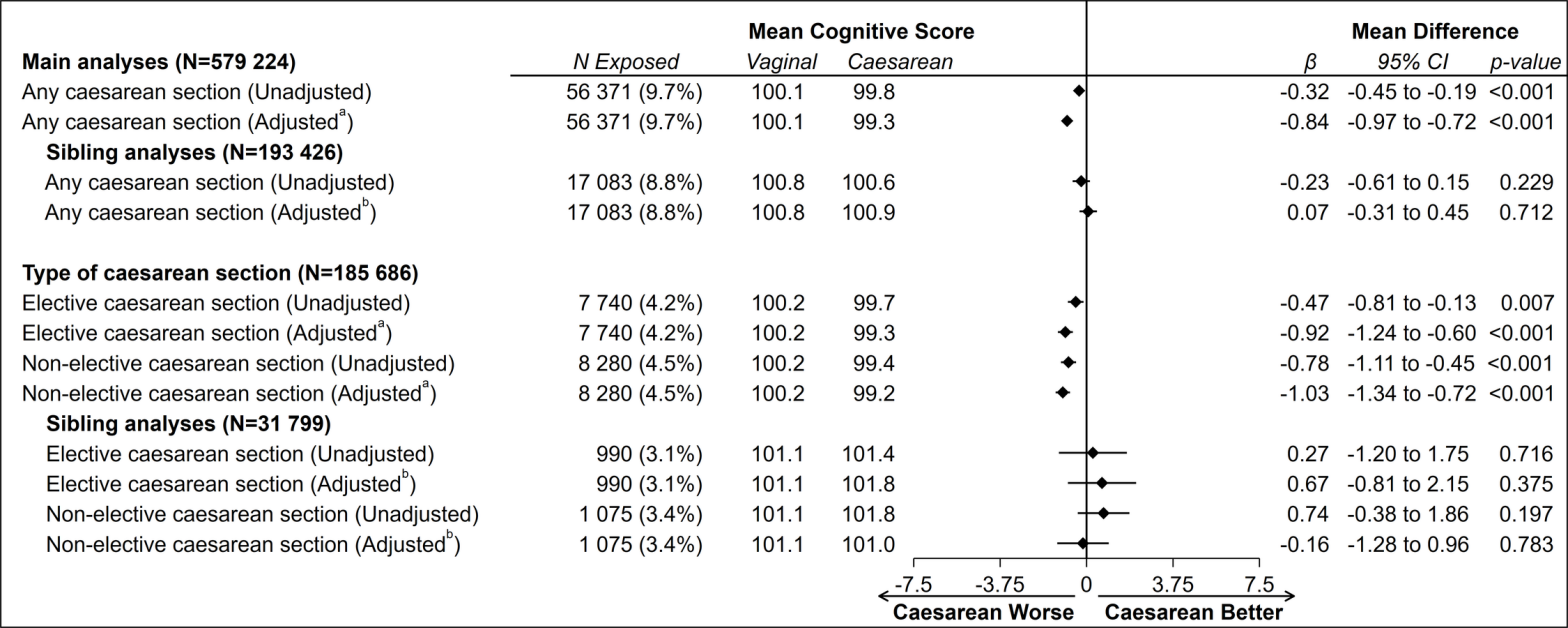
Mean score on the general cognitive ability assessment and the mean difference in the score between those born by caesarean section and vaginal delivery. ^a^Adjusted for maternal age, pre-eclampsia, hypertension and diabetes mellitus, family disposable income, parental country of birth, highest parental education, parental labour market position, offspring year of birth, parity, birth weight standardised by gestational age, and gestational age. ^b^Adjusted for same as above excluding family disposable income, parental country of birth, highest parental education and parental labour market position.

Those born by caesarean section had lower mean general cognitive ability, compared with those born vaginally, both before (mean score 99.8 vs 100.1, mean difference −0.32, 95% CI −0.45 to −0.19, p<0.001) and after accounting for confounding factors (mean score 99.3 vs 100.1, adjusted mean difference −0.84, 95% CI −0.97 to −0.72, p<0.001) (figure 2). There was no difference between elective and non-elective caesarean section’s relationship to general cognitive ability; general cognitive ability was lower among both elective (mean score 99.3 vs 100.2, adjusted mean difference −0.92, 95% CI −1.24 to −0.60, p<0.001) and non-elective caesarean section (mean score 99.2 vs 100.2, adjusted mean difference −1.03, 95% CI −1.34 to −0.72, p<0.001), as compared with those born vaginally.

However, when accounting for factors shared between brothers (genetic and environmental) (online supplemental table 2) the relationship between any type of caesarean section and general cognitive ability fully attenuated to the null (mean score 100.9 vs 100.8, adjusted mean difference 0.07, 95% CI −0.31 to 0.45, p=0.712) (figure 2). Similarly, both those born by elective (mean score 101.8 vs 100.1, adjusted mean difference 0.67, 95% CI −0.81 to 2.15, p=0.375) and non-elective (mean score 101.0 vs 101.1, adjusted mean difference −0.16, 95% CI −1.28 to 0.96, p=0.783) caesarean section had similar scores of general cognitive ability as compared with those born vaginally, after accounting for factors shared between brothers.

### Explorative analyses

**Figure 3:**
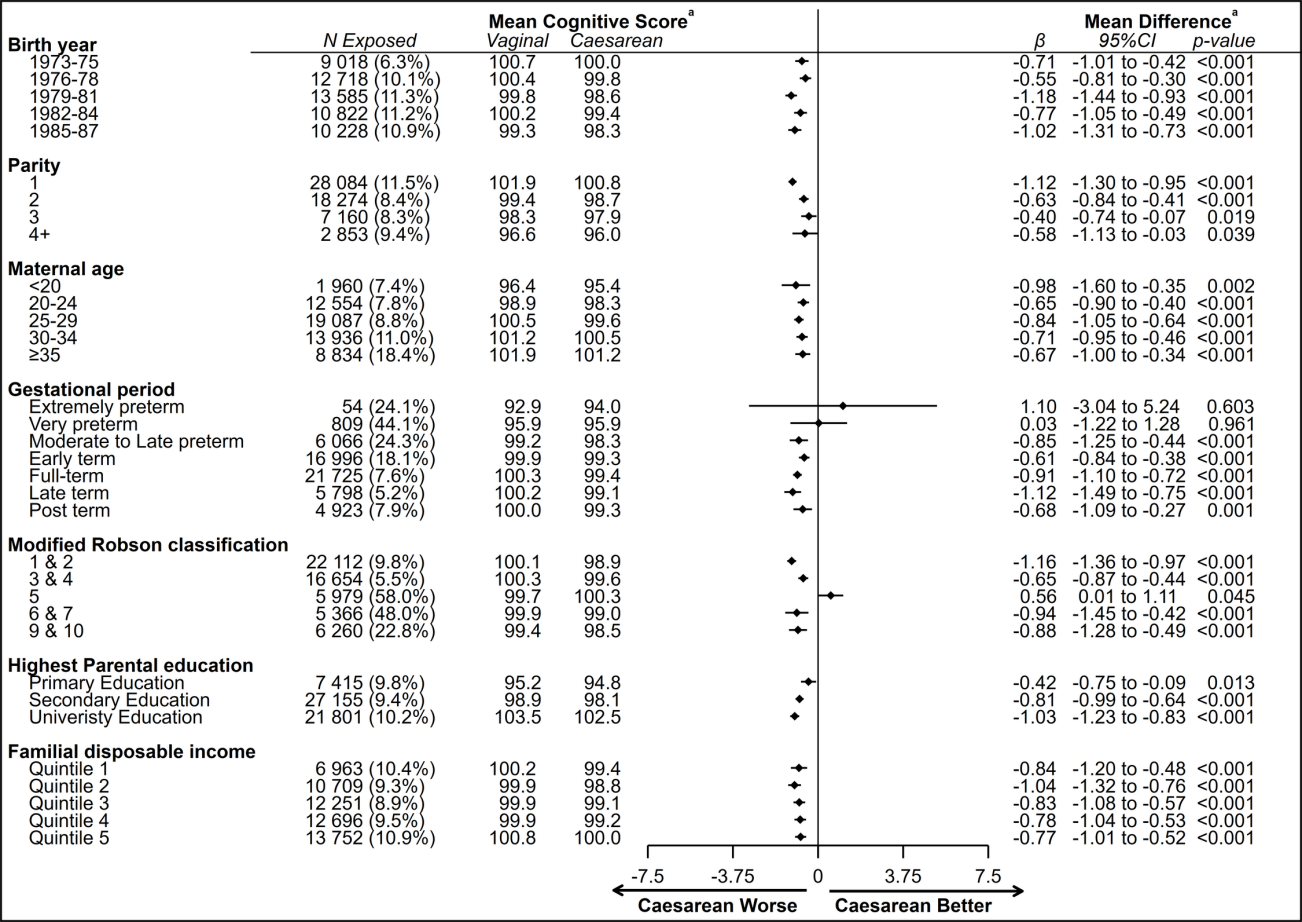
Mean score on the general cognitive ability assessment and the mean difference in the score between those born by caesarean section and vaginal delivery, stratified by birth year, parity, maternal age, gestational periods, modified Robson classification, highest parental education and familial disposable income. ^a^Adjusted for maternal age, pre-eclampsia, hypertension and diabetes mellitus, family disposable income, parental country of birth, highest parental education, parental labour market position, offspring year of birth, parity, birth weight standardised by gestational age and gestational age.

There were no consistent differences between our main cohort and when stratifying our analysis by gestational periods, birth year, parity, maternal age, modified Robson groups, parental education and parental income quintiles. Specifically, there was a persistent association between caesarean section and lower general cognitive ability in each stratum, after controlling for confounders (figure 3). The exception being in Robson class 5 (previous caesarean, cephalic, term), where birth by caesarean section was associated with an 0.56 (95% CI 0.01 to 1.11, p=0.045) units higher mean score as compared with those born vaginally.

We observed a similar pattern of association, although with smaller sample sizes, when considering elective and non-elective caesarean section in different strata, after controlling for confounders (online supplemental table 4). The greatest mean difference between elective and non-elective caesarean section was 1.99 units in the post-term born (95% CI −4.60 to 0.62, p=0.135), in favour of those born by non-elective caesarean section.

### Sensitivity analyses

No sensitivity analyses distinctly deviated from our main analyses. First, there was no difference in the association between caesarean section and general cognitive ability by those conscripts who performed the SEB80 battery and those who performed the CAT-SEB (Difference in adjusted β 0.25, 95% CI −0.00 to 0.50, p=0.054) (online supplemental table 5). There was consistency in the association between caesarean section and the components of both the SEB80 and the CAT-SEB battery (online supplemental table 6).

Second, there was a consistent relationship between caesarean section and prematurity (adjusted OR 3.33, 95% CI 3.23 to 3.43, p<0.001), with a stronger association in sibling analysis (online supplemental table 7). Third, there was no difference between our main analyses and when treating instrumental vaginal deliveries as a separate exposure (online supplemental table 8).

Forth, controlling for a history of caesarean section in the biological mother, maternal smoking, early pregnancy BMI and gestational weight gain z-score, among those where such information was available, was consistent with our main analyses (online supplemental table 9).

Fifth, not controlling for gestational age or birth weight z-score, as putative colliders (online supplemental figure 1), did not alter our findings (online supplemental table 10). Finally, excluding those born preterm and restricting to those born at-term was consistent with our main analyses (online supplemental table 11).

## Discussion

### Main findings

We present the to date largest examination of the role of caesarean section in offspring general cognitive ability. Consistent with previous studies, we found that those born by caesarean section have a lower general cognitive ability. However, the small magnitude of the association and the inconsistency in sibling analysis suggest that this relationship is of little clinical relevance and is influenced by confounding factors shared between siblings. Further, we did not observe any difference between elective and non-elective caesarean section in their relationship to general cognitive ability, contrary to what is implied by the absence of a beneficial stress mechanism among those born by elective caesarean section.^19^

### Comparison with previous studies

To the best of our knowledge, the previously largest study of caesarean section and offspring cognitive ability is an Australian study that separated types of caesarean section (N=153 730, N caesarean section=43 349).^15^ They found, consistent with our results, that there was a weak association between all types of caesarean section and school-aged children being classified as ‘developmentally high risk’ (10th percentile in two domains).^15^ Although not performing sibling analyses, the Australian study isolated low-risk pregnancies in efforts to minimise confounding by indication,^15^ where they observed a consistent risk in prelabour caesarean section and caesarean section following induction of labour, but not among caesarean section following spontaneous onset of labour.

Similar to our findings, although phenotypically different from general cognitive ability, another large-scale Swedish study observed that children born by caesarean section have slightly poorer primary school performance.^s53^ As the authors cautiously note, although both elective and non-elective caesarean section was associated with poorer performance, non-elective caesarean section was associated with the greatest odds of poor performance (OR 1.12, 95% CI 1.09 to 1.15) .^s53^ However, even their greatest estimate could easily be explained by residual confounding. Similarly, a recent New Zealand study found that caesarean section had a weak negative association with offspring educational performance in standard analyses, but that the relationship could be explained by confounders shared within families.^17^

Despite some authors arguing that the observed association between caesarean section and childhood cognitive development is causal,^s54^ we would like to propose that the nature of confounding by indication, which is inherently present in caesarean section epidemiological studies, will severely limit any conventional confounding control. Specifically, as others have also described for intrauterine influences,^s55^ we believe that the magnitude and variety of sources of confounding cannot be addressed in caesarean section epidemiology without, as we have attempted, employing methodology robust to unobserved confounding or conducting causal triangulation.^s56^

### Strengths and limitations

There are several strengths of our study as compared with previous work. Specifically, to the best of our knowledge, we have presented the largest investigation of caesarean section and cognitive ability. We have been able to extensively control for a wide range of observed confounders in, to a large extent, a representative sample of the male Swedish population. As the first study, we have been able to control for unobserved confounders shared within families in the relationship between caesarean section and cognitive ability—greatly increasing our ability to scrutinise the causality of the proposed relationship. Through the utilisation of national registries, we could ascertain prospective collection of data which ensures that our findings are not influenced by maternal recall bias. Furthermore, we have studied general cognitive ability using a standardised test, contrary to previous studies that have relied on proxy measures of cognitive ability^12^—which often are phenotypically different from assessed cognitive ability (eg, parent perception of offspring cognitive ability). Similarly, our examination of general cognitive ability does not limit our findings to clinical manifestation of morbidity, contrary to studies of caesarean section and diagnosed neurodevelopmental disorders.^s57^ Finally, the large population-based sample enabled us to highlight the consistency of our findings across a large set of exploratory stratified analyses.

There are several important limitations that need to be addressed. First, although the rate of caesarean section in our main cohort does not differ from that of the whole of Sweden during the same period (online supplemental table 12), there is a difference between our study (9.7%) and previous studies, such as that of the Australian study (28.2%).^15^ Differences in the rate of caesarean section between our study and other studies might influence our generalisability, but only if any factor that is different between populations also modifies the association between caesarean section and offspring cognitive ability. Importantly, our findings are largely consistent with that of the Australian study,^15^ highlighting that our findings may be robust across populations with different caesarean section rates.

Second, although we have employed sibling analyses in efforts to control for unobserved confounding, there are limitations of such designs. Specifically, sibling analyses are vulnerable to non-shared confounding,^s58^ measurement error,^s58^ carry-over effects^s59^ and shared mediator control.^25^ For example, random measurement error in the cognitive assessments, possibly due to stressors present at the testing facility, will bias sibling analyses estimates towards the null to a greater extent than conventional analyses. Similarly, conditioning on shared mediators will yield an underestimate of the total causal effect.^25^ As such, caution is warranted when directly interpreting effect estimates in sibling analyses.

Third, although we have retained a large proportion of the birth cohort (76%), selection into conscription and cognitive assessments likely introduce selection bias. In efforts to address this, we analysed prematurity as a positive control outcome within the main sample of conscripts, under the assumptions that caesarean section has a causal effect on prematurity and that healthy participant would bias such a relationship towards null (as expected for cognitive ability). Contrary to a selection bias resulting in null associations, we identified an association between caesarean section and prematurity—suggesting the absence of bias from selection (under the above assumptions). Despite a consistent association with the positive control outcome, the assumptions may have been violated, and selection bias may have attenuated our estimates of the relationship between caesarean section and general cognitive ability.

Fourth, our findings may not be directly generalisable to that of the commonly used Wechsler Adult Intelligence Scale.^s60^ However, the conscription batteries have been thoroughly evaluated,^27 28^ and the CAT-SEB battery positively correlate with performance on the Swedish Scholastic Aptitude Test^29^—similar to what would be expected of the Wechsler scale.

Finally, as we have only studied male conscripts, our findings may only apply to males. Although we see limited reasons for any sex-specific mechanisms, we cannot rule out such possibilities and large-scale studies with equiproportional female cohorts are needed to replicate our results.

### Clinical implications

The surging rates of caesarean sections have caused great concern, especially as caesarean section may carry long-term effects on offspring health. However, as noted in the 2018 Lancet series on Optimising caesarean section,^11^ it has been understudied whether birth by caesarean section has a long-term effect on offspring cognitive outcomes. While further large-scale studies are urgently needed, our findings that caesarean section is not a clinically relevant determinant of offspring cognitive ability is at least reassuring.

We are especially reassured as cognitive ability is a strong determinant of future health.^13^ For example, a 15-unit difference in the cognitive score would translate to a 1.25 HR of all-cause mortality—independently of other important determinants of health (eg, blood pressure, BMI and cigarette smoking).^13^ Fortunately, the largest difference we observed was −1.03 units, when comparing non-elective caesarean section to vaginal delivery—a difference of trivial relevance.

Accordingly, although further longitudinal studies are warranted, especially including female participants, clinicians and gravidas ought not to be concerned that the choice of mode of delivery will impact offspring cognitive ability.

## Conclusions

Birth by caesarean section is weakly associated with a lower general cognitive ability in young adult males. However, the magnitude of this association is not clinically relevant and seems to be largely explained by familial factors shared between siblings. Therefore, caesarean section may not represent an important determinant of offspring cognitive ability.

10.1136/ebmental-2021-300307.supp2 Supplementary data

## Data Availability

Data may be obtained from a third party and are not publicly available.
