# Supplementary material for: Caesarean section and its relationship to offspring general cognitive ability: a registry-based cohort study of half a million young male adults
Source: Evid Based Ment Health. 2021 Sep 12;25(1):7–14. doi: 10.1136/ebmental-2021-300307 (PMC8788259; doi:10.1136/ebmental-2021-300307)
Supplement: Supplementary data [file ebmental-2021-300307supp001.pdf]

Supplementary Table 1. Descriptive characteristics of the stepwise excluded cohort.

	Exclusion because of non-conscription			Exclusion because of no cognitive assessment (or duplicate value n=40)		
	Excluded	Eligible for inclusion	p-value[a]	Excluded	Main analytic sample	p-value[a]
Total number of observations, (%)	78554	638966		138296	579224	
Born by caesarean section, n (%)	10210 (13.0)	62778 (9.8)	<0.001	16617 (12.0)	56371 (9.7)	<0.001
General cognitive ability, mean (SD)	-	-	-	-	-	
Age at conscription (years), median (IQR)	-	-	-	18.3 (18.2, 18.6)	18.3 (18.1, 18.5)	<0.001
Birth weight (grams), mean (SD)	3454.5 (680.1)	3575.1 (540.9)	<0.001	3492.1 (635.1)	3578.6 (538.1)	<0.001
Foetal Growth, No (%)			<0.001			<0.001
Small for gestational age (<10th %ile)	9263 (11.8)	62424 (9.8)		15565 (11.3)	56122 (9.7)	
Appropriate for gestational age	61484 (78.3)	511617 (80.1)		108872 (78.7)	464229 (80.1)	
Large for gestational age (>90th %ile)	7807 (9.9)	64925 (10.2)		13859 (10.0)	58873 (10.2)	
Gestational age (weeks), mean (SD)	39.0 (2.6)	39.6 (1.8)	<0.001	39.2 (2.3)	39.6 (1.8)	<0.001
Gestational periods, No (%)			<0.001			<0.001
Extremely preterm (<28 wGA)	750 (1.0)	279 (<1)		805 (0.6)	224 (<1)	
Very preterm (28-31 wGA)	1223 (1.6)	2107 (0.3)		1494 (1.1)	1836 (0.3)	
Moderate to Late Preterm (32-36 wGA)	5328 (6.8)	28150 (4.4)		8459 (6.1)	25019 (4.3)	
Early term (37-38 wGA)	14940 (19.0)	104967 (16.4)		25894 (18.7)	94013 (16.2)	
Full-term (39-40 wGA)	36779 (46.8)	314418 (49.2)		66319 (48.0)	284878 (49.2)	
Late term (41 wGA)	12846 (16.4)	121179 (19.0)		23229 (16.8)	110796 (19.1)	
Post term (≥42 wGA)	6688 (8.5)	67866 (10.6)		12096 (8.7)	62458 (10.8)	
Modified Robson classification, No (%)			<0.001			<0.001
1+2	27918 (35.5)	247532 (38.7)		49774 (36.0)	225676 (39.0)	
3+4	39967 (50.9)	336418 (52.7)		71747 (51.9)	304638 (52.6)	
5	2017 (2.6)	11820 (1.8)		3534 (2.6)	10303 (1.8)	
6+7	1868 (2.4)	12314 (1.9)		3013 (2.2)	11169 (1.9)	
9+10	6784 (8.6)	30882 (4.8)		10228 (7.4)	27438 (4.7)	
Parity, median (IQR)	2.0 (1.0, 2.0)	2.0 (1.0, 2.0)	<0.001	2.0 (1.0, 2.0)	2.0 (1.0, 2.0)	<0.001
Maternal age at birth (years), mean (SD)	28.0 (5.3)	27.7 (5.0)	<0.001	28.0 (5.2)	27.6 (5.0)	<0.001
Categories of maternal age, No (%)			<0.001			<0.001
<20 years	3646 (4.6)	28907 (4.5)		6086 (4.4)	26467 (4.6)	
20-24 years	20966 (26.7)	175442 (27.5)		36350 (26.3)	160058 (27.6)	
25-29 years	27362 (34.8)	239170 (37.4)		49002 (35.4)	217530 (37.6)	
30-34 years	18023 (22.9)	141044 (22.1)		31987 (23.1)	127080 (21.9)	
≥35 years	8557 (10.9)	54403 (8.5)		14871 (10.8)	48089 (8.3)	
Maternal diabetes mellitus, No (%)	564 (0.7)	2944 (0.5)	<0.001	943 (0.7)	2565 (0.4)	<0.001
Maternal hypertension, No (%)	185 (0.2)	750 (0.1)	<0.001	321 (0.2)	614 (0.1)	<0.001
Preeclampsia, No (%)	1034 (1.3)	4192 (0.7)	<0.001	1743 (1.3)	3483 (0.6)	<0.001
Highest parental education, No (%)			<0.001			<0.001
Primary Education	15401 (19.6)	83770 (13.1)		23838 (17.2)	75333 (13.0)	
Secondary Education	40470 (51.5)	320235 (50.1)		70717 (51.1)	289988 (50.1)	
University Education	22683 (28.9)	234961 (36.8)		43741 (31.6)	213903 (36.9)	
Familial disposable income, No (%)			<0.001			<0.001
Quintile 1	17165 (21.9)	77319 (12.1)		27347 (19.8)	67137 (11.6)	
Quintile 2	17828 (22.7)	128307 (20.1)		31291 (22.6)	114844 (19.8)	
Quintile 3	16001 (20.4)	150713 (23.6)		29537 (21.4)	137177 (23.7)	
Quintile 4	14394 (18.3)	145327 (22.7)		26374 (19.1)	133347 (23.0)	
Quintile 5	13166 (16.8)	137300 (21.5)		23747 (17.2)	126719 (21.9)	
Parent(s) born in Sweden, No (%)			<0.001			<0.001
Both parents	53446 (68.0)	550494 (86.2)		103237 (74.6)	500703 (86.4)	
One parent	11921 (15.2)	61838 (9.7)		18562 (13.4)	55197 (9.5)	
Neither parent	13187 (16.8)	26634 (4.2)		16497 (11.9)	23324 (4.0)	
Parental labour market position, No (%)			<0.001			<0.001
Others	6793 (8.6)	24844 (3.9)		9833 (7.1)	21804 (3.8)	
Unskilled workers	17539 (22.3)	113988 (17.8)		28830 (20.8)	102697 (17.7)	
Skilled workers	16035 (20.4)	123327 (19.3)		27626 (20.0)	111736 (19.3)	
Self-employed and farmers	4362 (5.6)	39077 (6.1)		7784 (5.6)	35655 (6.2)	
Non-manual workers at lower level	9814 (12.5)	95520 (14.9)		18436 (13.3)	86898 (15.0)	
Non-manual workers at intermediate level	14484 (18.4)	153104 (24.0)		27513 (19.9)	140075 (24.2)	
Non-manual workers at higher level	9527 (12.1)	89106 (13.9)		18274 (13.2)	80359 (13.9)	

[a]Calculated using χ2 test (categorical), t-test (continuous), and Wilcoxon rank-sum test (continuous skewed).
Abbreviations: IQR, interquartile range; No., number of observations; SD, standard deviation; wGA, weeks of gestational age; %ile, percentile.

Supplementary Table 2. Descriptive characteristics of the siblings in the main cohort and the sub-cohort with detailed delivery data, stratified by mode of delivery.

	Main Cohort		Sub-cohort with detailed delivery data		
	Vaginal Delivery	Any Caesarean Section	Vaginal Delivery	Elective Caesarean Section	Non-elective Caesarean Section
Total number of observations, (%)	176343 (91.2)	17083 (8.8)	29734 (93.5)	990 (3.1)	1075 (3.4)
Age at conscription (years), median (IQR)	18.3 (18.1, 18.5)	18.3 (18.1, 18.5)	18.3 (18.1, 18.5)	18.3 (18.1, 18.5)	18.3 (18.1, 18.5)
Birth weight (grams), mean (SD)	3625.1 (507.8)	3412.3 (651.6)	3641.1 (500.1)	3446.9 (543.2)	3394.6 (758.0)
Foetal Growth, No (%)					
Small for gestational age (<10th %ile)	14920 (8.5)	2051 (12.0)	1949 (6.6)	73 (7.4)	117 (10.9)
Appropriate for gestational age	142452 (80.8)	13066 (76.5)	24268 (81.6)	783 (79.1)	816 (75.9)
Large for gestational age (>90th %ile)	18971 (10.8)	1966 (11.5)	3517 (11.8)	134 (13.5)	142 (13.2)
Gestational age (weeks), mean (SD)	39.7 (1.6)	38.7 (2.2)	39.5 (1.5)	38.1 (1.4)	38.6 (2.9)
Gestational periods, No (%)					
Extremely preterm (<28 wGA)	35 (<1)	20 (0.1)	<10	<10	<10
Very preterm (28-31 wGA)	246 (0.1)	199 (1.2)	25 (0.1)	<10	22 (2.0)
Moderate to Late Preterm (32-36 wGA)	5710 (3.2)	1654 (9.7)	1043 (3.5)	61 (6.2)	177 (16.5)
Early term (37-38 wGA)	25707 (14.6)	5536 (32.4)	5018 (16.9)	610 (61.6)	213 (19.8)
Full-term (39-40 wGA)	89973 (51.0)	6671 (39.1)	16122 (54.2)	270 (27.3)	371 (34.5)
Late term (41 wGA)	35635 (20.2)	1641 (9.6)	5494 (18.5)	30 (3.0)	174 (16.2)
Post term (≥42 wGA)	19037 (10.8)	1362 (8.0)	2031 (6.8)	15 (1.5)	114 (10.6)
Modified Robson classification, No (%)					
1+2	59596 (33.8)	5233 (30.6)	9529 (32.0)	163 (16.5)	480 (44.7)
3+4	105065 (59.6)	3571 (20.9)	18154 (61.1)	161 (16.3)	197 (18.3)
5	3528 (2.0)	4978 (29.1)	746 (2.5)	498 (50.3)	128 (11.9)
6+7	1764 (1.0)	1614 (9.4)	159 (0.5)	106 (10.7)	94 (8.7)
9+10	6390 (3.6)	1687 (9.9)	1146 (3.9)	62 (6.3)	176 (16.4)
Parity, median (IQR)	2.0 (1.0, 2.0)	2.0 (1.0, 2.0)	2.0 (1.0, 2.0)	2.0 (1.0, 3.0)	1.0 (1.0, 2.0)
Maternal age at birth (years), mean (SD)	27.4 (4.6)	28.6 (5.1)	27.8 (4.4)	29.6 (4.8)	27.8 (4.5)
Categories of maternal age, No (%)					
<20 years	6808 (3.9)	485 (2.8)	611 (2.1)	<10	25 (2.3)
20-24 years	51330 (29.1)	3911 (22.9)	7829 (26.3)	174 (17.6)	280 (26.0)
25-29 years	69505 (39.4)	6411 (37.5)	12433 (41.8)	385 (38.9)	449 (41.8)
30-34 years	37783 (21.4)	4266 (25.0)	6946 (23.4)	270 (27.3)	243 (22.6)
≥35 years	10917 (6.2)	2010 (11.8)	1915 (6.4)	156 (15.8)	78 (7.3)
Maternal diabetes mellitus, No (%)	494 (0.3)	225 (1.3)	132 (0.4)	15 (1.5)	11 (1.0)
Maternal hypertension, No (%)	128 (0.1)	45 (0.3)	47 (0.2)	<10	<10
Preeclampsia, No (%)	695 (0.4)	241 (1.4)	330 (1.1)	26 (2.6)	56 (5.2)
Highest parental education, No (%)					
Primary Education	20299 (11.5)	1977 (11.6)	2604 (8.8)	78 (7.9)	87 (8.1)
Secondary Education	85351 (48.4)	7904 (46.3)	14639 (49.2)	481 (48.6)	533 (49.6)
University Education	70693 (40.1)	7202 (42.2)	12491 (42.0)	431 (43.5)	455 (42.3)
Familial disposable income, No (%)					
Quintile 1	18790 (10.7)	1969 (11.5)	6083 (20.5)	194 (19.6)	247 (23.0)
Quintile 2	34737 (19.7)	3155 (18.5)	7663 (25.8)	209 (21.1)	265 (24.7)
Quintile 3	41380 (23.5)	3781 (22.1)	6601 (22.2)	214 (21.6)	238 (22.1)
Quintile 4	42455 (24.1)	3915 (22.9)	5249 (17.7)	183 (18.5)	182 (16.9)
Quintile 5	38981 (22.1)	4263 (25.0)	4138 (13.9)	190 (19.2)	143 (13.3)
Parent(s) born in Sweden, No (%)					
Both parents	156264 (88.6)	15030 (88.0)	25930 (87.2)	846 (85.5)	947 (88.1)
One parent	14397 (8.2)	1450 (8.5)	2583 (8.7)	114 (11.5)	84 (7.8)
Neither parent	5682 (3.2)	603 (3.5)	1221 (4.1)	30 (3.0)	44 (4.1)
Parental labour market position, No (%)					
Others	5776 (3.3)	553 (3.2)	1168 (3.9)	42 (4.2)	38 (3.5)
Unskilled workers	29274 (16.6)	2691 (15.8)	4516 (15.2)	152 (15.4)	175 (16.3)
Skilled workers	33544 (19.0)	3046 (17.8)	5726 (19.3)	165 (16.7)	214 (19.9)
Self-employed and farmers	12207 (6.9)	1191 (7.0)	1950 (6.6)	56 (5.7)	73 (6.8)
Non-manual workers at lower level	24456 (13.9)	2382 (13.9)	3858 (13.0)	146 (14.7)	138 (12.8)
Non-manual workers at intermediate level	44432 (25.2)	4300 (25.2)	7457 (25.1)	223 (22.5)	254 (23.6)
Non-manual workers at higher level	26654 (15.1)	2920 (17.1)	5059 (17.0)	206 (20.8)	183 (17.0)

Abbreviations: IQR, interquartile range; No., number of observations; SD, standard deviation; wGA, weeks of gestational age; %ile, percentile.

Supplementary Table 3. Description of the original and modified Robson classification

Description[a]	Original Robson classification	Modified Robson classification
Nulliparous women with a single cephalic pregnancy, ≥37 weeks gestation in spontaneous labour	1	1+2
Nulliparous women with a single cephalic pregnancy, ≥37 weeks gestation who had labour induced or were delivered by caesarean section before labour	2	
Multiparous women without a previous caesarean section, with a single cephalic pregnancy, ≥37 weeks gestation in spontaneous labour	3	3+4
Multiparous women without a previous caesarean section, with a single cephalic pregnancy, ≥37 weeks gestation who had labour induced or were delivered by caesarean section before labour	4	
All multiparous women with at least one previous caesarean section, with a single cephalic pregnancy, ≥37 weeks gestation	5	5
All nulliparous women with a single breech pregnancy	6	6+7
All multiparous with a single breech (including previous caesarean section)	7	
All multiparous women with a single breech pregnancy including women with previous caesarean section	8	N/A
All women with a single pregnancy with a transverse or oblique lie, including women with previous caesarean section	9	9+10
All women with a single cephalic pregnancy < 37 weeks gestation, including women with previous caesarean section	10	

[a]As reported in the World Health Organization Robson Classification: Implementation Manual (ISBN: 978-92-4-151319-7, 2017)

Supplementary Table 4. Elective and non-elective caesarean section and offspring general cognitive ability, by birth year, parity, maternal age, gestational periods, modified Robson classification, highest parental education and familial disposable income.

	Mean Cognitive core					Mean Difference from Vaginal Delivery					
	Vaginal	Elective Caesarean Section		Non-elective Caesarean Section		Elective Caesarean Section			Non-elective Caesarean Section		
	Mean	N Exposed	Mean	N exposed	Mean	β	95% CI	p-value	β	95% CI	p-value
Birth year											
1982-84	100.6	3,927 (4.2%)	99.7	4,145 (4.4%)	99.9	-0.86	-1.32 to -0.41	<0.001	-0.76	-1.19 to -0.32	0.001
1985-87	99.8	3,813 (4.2%)	98.9	4,135 (4.5%)	98.7	-0.91	-1.36 to -0.45	<0.001	-1.19	-1.62 to -0.75	<0.001
Parity											
1	101.9	2,423 (3.2%)	100.6	5,009 (6.6%)	100.7	-1.27	-1.85 to -0.69	<0.001	-1.17	-1.58 to -0.77	<0.001
2	99.6	3,244 (4.8%)	98.7	2,021 (3%)	98.8	-0.92	-1.41 to -0.43	<0.001	-0.77	-1.37 to -0.17	0.012
3	98.6	1,537 (5%)	98	865 (2.8%)	97.7	-0.66	-1.38 to 0.06	0.071	-0.90	-1.84 to 0.04	0.060
4+	97.2	536 (4.8%)	97.2	385 (3.5%)	96.2	0.05	-1.14 to 1.24	0.934	-0.96	-2.43 to 0.51	0.202
Maternal age											
<20	96.3	98 (1.8%)	94.5	235 (4.4%)	96	-1.76	-4.70 to 1.18	0.241	-0.34	-2.12 to 1.44	0.706
20-24	98.8	1,133 (2.6%)	98.2	1,972 (4.5%)	97.8	-0.66	-1.48 to 0.16	0.114	-1.01	-1.64 to -0.39	0.001
25-29	100.4	2,538 (3.7%)	99.5	2,977 (4.3%)	99.3	-0.93	-1.49 to -0.37	0.001	-1.14	-1.65 to -0.62	<0.001
30-34	101.1	2,260 (4.9%)	100.6	1,968 (4.2%)	100.2	-0.53	-1.12 to 0.07	0.083	-0.89	-1.52 to -0.25	0.006
≥35	101.6	1,711 (8.3%)	100.5	1,128 (5.5%)	100.7	-1.07	-1.77 to -0.38	0.002	-0.90	-1.74 to -0.06	0.036
Gestational period											
Extremely preterm (<28 wGA)	91.3	<10	-	29 (35.8%)	93.8	-	-	-	-	-	-
Very preterm (28-31 wGA)	96.3	42 (7.4%)	92.9	277 (48.9%)	94.8	-3.40	-7.85 to 1.05	0.134	-1.52	-3.83 to 0.79	0.197
Moderate to Late Preterm (32-36 wGA)	99.7	506 (5.9%)	99.1	1,424 (16.7%)	98.5	-0.62	-1.91 to 0.67	0.346	-1.16	-1.95 to -0.38	0.004
Early term (37-38 wGA)	100.1	4,723 (13.5%)	99.2	1,589 (4.5%)	99.3	-0.85	-1.29 to -0.42	<0.001	-0.76	-1.46 to -0.07	0.032
Full-term (39-40 wGA)	100.4	2,116 (2.2%)	99.3	2,672 (2.8%)	99.6	-1.12	-1.72 to -0.53	<0.001	-0.80	-1.34 to -0.27	0.003
Late term (41 wGA)	100.3	209 (0.6%)	99.5	1,337 (4%)	99.1	-0.80	-2.70 to 1.10	0.411	-1.19	-1.95 to -0.43	0.002
Post term (≥42 wGA)	100	143 (1.0%)	97.6	952 (6.9%)	99.6	-2.32	-4.79 to 0.14	0.064	-0.33	-1.26 to 0.60	0.484
Modified Robson classification											
1 & 2	100.3	1,755 (2.5%)	98.7	3,912 (5.6%)	99.3	-1.58	-2.25 to -0.90	<0.001	-1.02	-1.48 to -0.56	<0.001
3 & 4	100.2	2,620 (2.7%)	99.5	1,791 (1.8%)	99.6	-0.75	-1.29 to -0.21	0.006	-0.65	-1.30 to -0.01	0.047
5	99.6	2,068 (39.6%)	99.5	440 (8.4%)	99.6	-0.14	-0.94 to 0.67	0.743	-0.07	-1.44 to 1.30	0.923
6 & 7	100	781 (33%)	99.3	601 (25.4%)	99.4	-0.75	-2.05 to 0.55	0.258	-0.66	-2.00 to 0.68	0.336
9 & 10	100	516 (5.5%)	99	1,536 (16.5%)	98.3	-0.94	-2.21 to 0.34	0.151	-1.70	-2.46 to -0.94	<0.001
Highest Parental education											
Primary Education	95.2	810 (4.3%)	94.7	890 (4.7%)	94.3	-0.46	-1.41 to 0.48	0.336	-0.90	-1.80 to 0.00	0.051
Secondary Education	98.8	3,721 (3.9%)	97.9	4,257 (4.5%)	97.8	-0.85	-1.31 to -0.40	<0.001	-0.95	-1.38 to -0.52	<0.001
University Education	103.5	3,209 (4.5%)	102.4	3,133 (4.3%)	102.3	-1.11	-1.62 to -0.60	<0.001	-1.18	-1.68 to -0.68	<0.001
Familial disposable income											
Quintile 1	100.2	1,445 (3.7%)	99	1,829 (4.7%)	99.6	-1.24	-1.98 to -0.50	0.001	-0.62	-1.30 to 0.06	0.072
Quintile 2	99.9	1,709 (3.7%)	98.8	2,056 (4.5%)	98.8	-1.09	-1.77 to -0.41	0.002	-1.10	-1.72 to -0.49	<0.001
Quintile 3	99.9	1,647 (4.1%)	99.2	1,814 (4.5%)	98.9	-0.74	-1.43 to -0.05	0.036	-1.01	-1.65 to -0.38	0.002
Quintile 4	100.2	1,481 (4.4%)	99.3	1,394 (4.2%)	98.8	-0.90	-1.64 to -0.17	0.015	-1.45	-2.18 to -0.71	<0.001
Quintile 5	101.4	1,458 (5.3%)	100.7	1,187 (4.3%)	100.3	-0.62	-1.36 to 0.11	0.097	-1.08	-1.90 to -0.26	0.010

Supplementary Table 5. The difference in the relationship between caesarean section and general cognitive ability by the type of test-battery that the conscript performed (SEB80 vs CAT-SEB).

	SEB80 (N=276 567)			CAT-SEB (N=302 657)					
	β	95% CI	p-value	β	95% CI	p-value	β-Difference	95% CI	p-value
Vaginal delivery	Ref.	-	-	Ref.	-	-			
Caesarean Section[a]	-0.70	-0.90 to -0.51	<0.001	-0.951	-1.11 to -0.79	<0.001	0.25	-0.00 to 0.50	0.054

[a]Adjusted for: maternal age, preeclampsia, hypertension, and diabetes mellitus, family disposable income, parental country of birth, parental labour market position, offspring year of birth, parity, birth weight standardized to gestational age and gestational age

Supplementary Table 6. The relationship between caesarean section and general cognitive ability and its components.

	N	β[a,b]	95% CI	P-value
**SEB80**				
G-factor	142 573	-0.93	-1.22 to -0.64	<0.001
Technical	142 573	-0.93	-1.24 to -0.63	<0.001
Logic	142 573	-0.69	-0.98 to -0.39	<0.001
Verbal	142 573	-0.69	-0.98 to -0.40	<0.001
Visuospatial	142 573	-0.85	-1.16 to -0.55	<0.001
**CAT-SEB**				
G-factor	299 749	-0.56	-0.74 to -0.39	<0.001
Spatial (General visualization)	299 749	-0.42	-0.59 to -0.26	<0.001
Verbal (Crystallized intelligence)	299 749	-0.96	-1.12 to -0.80	<0.001

[a]Mean difference as compared to those born vaginally

[b]Adjusted for: maternal age, preeclampsia, hypertension, and diabetes mellitus, family disposable income, parental country of birth, parental labour market position, offspring year of birth, parity and birth weight standardized to gestational age and gestational age

Supplementary Table 7. Sensitivity analysis using prematurity as a positive causal outcome.

Prematurity	N	Odds ratio[a]	95% CI	P-value
Conventional Crude	579 224	3.50	3.40 to 3.60	<0.001
Conventional Adjusted[b]	579 224	3.33	3.23 to 3.43	<0.001
Sibling-analysis Crude	13 892	5.94	5.11 to 6.92	<0.001
Sibling-analysis Adjusted[c]	13 892	7.43	6.28 to 8.79	<0.001

[a]As compared to those born vaginally
[b]Adjusted for: maternal age, preeclampsia, hypertension, and diabetes mellitus, family disposable income, parental country of birth, parental labour market position, offspring year of birth, parity and birth weight standardized to gestational age
[c]Adjusted for same as above excluding family disposable income, parental country of birth, highest parental education and parental labour market position

Supplementary Table 8. Sensitivity analysis of the relationship between caesarean section and general cognitive ability, as compared to vaginal delivery without instrumental support.

	N exposed	β[a]	95% CI	P-value
Vaginal delivery	483 603 (83.5%)	Ref.	-	-
Assisted vaginal delivery	39 250 (6.8%)	-0.35	-0.50 to -0.21	<0.001
Caesarean section	56 371 (9.7%)	-0.88	-1.00 to -0.75	<0.001

[a]Adjusted for: maternal age, preeclampsia, hypertension, and diabetes mellitus, family disposable income, parental country of birth, parental labour market position, offspring year of birth, parity and birth weight standardized to gestational age and gestational age

Supplementary Table 9. Sensitivity analysis of the relationship between caesarean section and general cognitive ability.

	N	β[a]	95% CI	P-value
Model 1[b]	579 224	-0.88	-1.01 to -0.75	<0.001
Model 2[c]	123 032	-0.98	-1.25 to -0.72	<0.001
Model 3[d]	123 032	-0.95	-1.21 to -0.69	<0.001
Model 4[e]	123 032	-0.87	-1.14 to -0.61	<0.001
Model 5[f]	123 032	-0.94	-1.20 to -0.67	<0.001
Model 6[g]	123 032	-0.79	-1.06 to -0.53	<0.001

[a]Mean difference as compared to those born vaginally
[b]Adjusted for confounders and further adjusted for history of caesarean sections.
[c]Main confounder adjusted analysis repeated in the subset of the cohort (for cross-reference) with available maternal smoking, early pregnancy BMI and gestational weight gain.
[d]Adjusted for confounders and further adjusted for maternal smoking.
[e]Adjusted for confounders and further adjusted for early pregnancy maternal BMI.
[f]Adjusted for confounders and further adjusted for gestational weight gain z-score.
[g]Adjusted for confounders and further jointly adjusted for maternal smoking, maternal early pregnancy BMI and gestational weight gain z-score.

Supplementary Table 10. Sensitivity analysis of the relationship between caesarean section and general cognitive ability, by gestational age adjustment and gestational age inclusion.

	β[a]	95% CI	P-value
Excluding GA adjustment			
Any CS	-0.97	-1.09 to -0.85	<0.001
Elective CS	-1.08	-1.40 to -0.76	<0.001
Non-elective CS	-1.15	-1.45 to -0.84	<0.001
Excluding BW adjustment			
Any CS	-0.94	-1.06 to -0.81	<0.001
Elective CS	-0.93	-1.26 to -0.61	<0.001
Non-elective CS	-1.11	-1.43 to -0.80	<0.001
Excluding GA and BW adjustment			
Any CS	-1.06	-1.18 to -0.94	<0.001
Elective CS	-1.10	-1.42 to -0.78	<0.001
Non-elective CS	-1.24	-1.54 to -0.93	<0.001

[a]Mean difference as compared to those born vaginally
[b]Adjusted for confounders excluding adjustment for gestational age.
[c]Adjusted for confounders excluding adjustment for birth weight.
[d]Adjusted for confounders excluding adjustment for birth weight and gestational age.

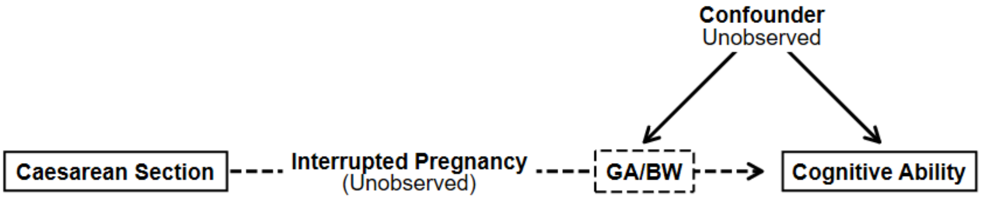

Supplementary Figure 1. The possible causal relationship between caesarean section and cognitive ability, whereby gestational age (GA) and birth weight (BW) acts as colliders because of a confounder (unobserved) and the iatrogenic effect that caesarean section has on the interruption of pregnancy (unobserved).

Supplementary Table 11. Sensitivity analysis of the relationship between caesarean section and general cognitive ability, by gestational age adjustment and gestational age inclusion.

	N exposed	β[a]	95% CI	p-value
Excluding those born preterm[b]				
Any CS	49 442 (8.9%)	-0.85	-0.98 to -0.72	<0.001
Elective CS	7 191 (4.1%)	-1.05	-1.39 to -0.72	<0.001
Non-elective CS	6 550 (3.7%)	-0.85	-1.20 to -0.51	<0.001
Restricted to those born at-term[c]				
Any CS	48 310 (8.9%)	-0.83	-0.97 to -0.70	<0.001
Elective CS	7 164 (4.1%)	-1.04	-1.37 to -0.70	<0.001
Non-elective CS	6 415 (3.7%)	-0.85	-1.20 to -0.50	<0.001

[a]Mean difference as compared to those born vaginally
[b]Adjusted for confounders and excluding those born preterm (<37 weeks).
[c]Adjusted for confounders restricted to those born at-term (37-42 weeks).

Supplementary Table 12. The difference in the rate of caesarean section between the Swedish Medical Birth Registry and the main cohort.

Year	Swedish Medical Birth Registry [a]	Main Cohort
1973	5.3%	5.3%
1974	6.6%	6.5%
1975	7.6%	7.3%
1976	9.4%	9.2%
1977	10.6%	10.2%
1978	11.3%	11.1%
1979	11.4%	11.0%
1980	11.7%	11.3%
1981	12.1%	11.7%
1982	11.9%	11.4%
1983	12.0%	11.1%
1984	11.5%	11.2%
1985	11.8%	11.4%
1986	11.3%	10.9%
1987	11.0%	10.3%

[a]Publicly available data from the National Board of Health and Welfare (https://www.socialstyrelsen.se/en/)
