# Supplementary material for: Caesarean section and its relationship to offspring general cognitive ability: a registry-based cohort study of half a million young male adults
Source: Evid Based Ment Health. 2021 Sep 12;25(1):7–14. doi: 10.1136/ebmental-2021-300307 (PMC8788259; doi:10.1136/ebmental-2021-300307)
Supplement: Supplementary data [file ebmental-2021-300307supp002.pdf]

Excess References

31 Myrskyla M, Silventoinen K, Tynelius P, et al. Is later better or worse? Association of advanced parental age with offspring cognitive ability among half a million young Swedish men. *Am J Epidemiol* 2013;177:649-55.

32 Pinheiro TV, Brunetto S, Ramos JG, et al. Hypertensive disorders during pregnancy and health outcomes in the offspring: a systematic review. *J Dev Orig Health Dis* 2016;7:391-407.

33 Penfield CA, Nageotte MP, Wing DA. Disparate Rates of Cesarean Delivery in Term Nulliparous Women with Hypertensive Disorders of Pregnancy. *Am J Perinatol* 2019;36:997-1001.

34 The HAPO Study Cooperative Research Group, Metzger BE, Lowe LP, et al. Hyperglycemia and adverse pregnancy outcomes. *N Engl J Med* 2008;358:1991-2002.

35 Fraser A, Nelson SM, Macdonald-Wallis C, et al. Associations of existing diabetes, gestational diabetes, and glycosuria with offspring IQ and educational attainment: the Avon Longitudinal Study of Parents and Children. *Exp Diabetes Res* 2012;2012:963735.

36 Lundborg P, Nilsson A, Rooth D-O. Parental Education and Offspring Outcomes: Evidence from the Swedish Compulsory School Reform. *American Economic Journal: Applied Economics* 2014;6:253-78.

37 Barclay KJ. A within-family analysis of birth order and intelligence using population conscription data on Swedish men. *Intelligence* 2015;49:134-43.

38 Menacker F, Declercq E, Macdorman MF. Cesarean delivery: background, trends, and epidemiology. *Semin Perinatol* 2006;30:235-41.

39 Bilder DA, Pinborough-Zimmerman J, Bakian AV, et al. Prenatal and perinatal factors associated with intellectual disability. *Am J Intellect Dev Disabil* 2013;118:156-76.

40 Lundgren M, Cnattingius S, Jonsson B, et al. Intellectual performance in young adult males born small for gestational age. *Growth Horm IGF Res* 2004;14 Suppl A:S7-8.

41 Yang S, Bergvall N, Cnattingius S, et al. Gestational age differences in health and development among young Swedish men born at term. *Int J Epidemiol* 2010;39:1240-9.

42 Allison PD. Fixed Effects Regression Models. Thousand Oaks, California. , 2009.

43 Spong CY. Defining "term" pregnancy: recommendations from the Defining "Term" Pregnancy Workgroup. *Jama* 2013;309:2445-6.

44 Pyykonen A, Gissler M, Lokkegaard E, et al. Cesarean section trends in the Nordic Countries - a comparative analysis with the Robson classification. *Acta Obstet Gynecol Scand* 2017;96:607-16.

45 Tilstra AM, Masters RK. Worth the Weight? Recent Trends in Obstetric Practices, Gestational Age, and Birth Weight in the United States. *Demography* 2020;57:99-121.

46 Tchetgen Tchetgen E. The control outcome calibration approach for causal inference with unobserved confounding. *Am J Epidemiol* 2014;179:633-40.

47 Braun JM, Daniels JL, Kalkbrenner A, et al. The effect of maternal smoking during pregnancy on intellectual disabilities among 8-year-old children. *Paediatr Perinat Epidemiol* 2009;23:482-91.

48 Lurie S, Ribenzaft S, Boaz M, et al. The effect of cigarette smoking during pregnancy on mode of delivery in uncomplicated term singleton pregnancies. *J Matern Fetal Neonatal Med* 2014;27:812-5.

49 Chu SY, Kim SY, Schmid CH, et al. Maternal obesity and risk of cesarean delivery: a meta-analysis. *Obes Rev* 2007;8:385-94.

50 Adane AA, Mishra GD, Tooth LR. Maternal pre-pregnancy obesity and childhood physical and cognitive development of children: a systematic review. *Int J Obes (Lond)* 2016;40:1608-18.

51 Gage SH, Lawlor DA, Tilling K, et al. Associations of maternal weight gain in pregnancy with offspring cognition in childhood and adolescence: findings from the Avon Longitudinal Study of Parents and Children. *Am J Epidemiol* 2013;177:402-10.

52 Johansson K, Hutcheon JA, Stephansson O, et al. Pregnancy weight gain by gestational age and BMI in Sweden: a population-based cohort study. *Am J Clin Nutr* 2016;103:1278-84.

53 Curran EA, Kenny LC, Dalman C, et al. Birth by caesarean section and school performance in Swedish adolescents- a population-based study. *BMC Pregnancy Childbirth* 2017;17:121.

54 Polidano C, Zhu A, Bornstein JC. The relation between cesarean birth and child cognitive development. *Sci Rep* 2017;7:11483.

55 Smith GD. Assessing intrauterine influences on offspring health outcomes: can epidemiological studies yield robust findings? *Basic Clin Pharmacol Toxicol* 2008;102:245-56.

56 Lawlor DA, Tilling K, Davey Smith G. Triangulation in aetiological epidemiology. *Int J Epidemiol* 2016;45:1866-86.

57 Curran EA, Dalman C, Kearney PM, et al. Association Between Obstetric Mode of Delivery and Autism Spectrum Disorder: A Population-Based Sibling Design Study. *JAMA Psychiatry* 2015;72:935-42.

58 Frisell T, Oberg S, Kuja-Halkola R, et al. Sibling comparison designs: bias from non-shared confounders and measurement error. *Epidemiology* 2012;23:713-20.

59 Sjolander A, Frisell T, Kuja-Halkola R, et al. Carryover Effects in Sibling Comparison Designs. *Epidemiology* 2016;27:852-8.

60 Wechsler D. Wechsler adult intelligence scale. 1955
